# QSAR analysis of drugs using graph based degree based topological indices and regression models

**DOI:** 10.1038/s41598-025-33504-7

**Published:** 2026-01-05

**Authors:** Zeeshan Saleem Mufti, Aqsa Kabeer, Abdulrahman A. Almehizia, Gamachu Adugna Ganati

**Affiliations:** 1 Department of Mathematics and Statistics, The University of Lahore, Lahore, Pakistan; 2 Drug Exploration and Development Chair (DEDC), Department of Pharmaceutical Chemistry, College of Pharmacy, King Saud University, Riyadh, 11451 Saudi Arabia; 3 Department of Mathematics, Wallaga University, Nekemte, Ethiopia; 4 School of Mathematics and Statistics, Linyi University, Linyi, 276005 P.R. China

**Keywords:** Chemistry, Mathematics and computing

## Abstract

Drugs are chemical solutions that are extensively used in diagnosing, prevention and treatment of diseases. To develop the drugs, it is important to understand the correlation between the drugs structure and their physicochemical behavior. Molecular network analysis is a systematic analysis of structural features, with topological indices having an important role in the measurement of molecular architecture. Ten popular topological indices, including Atom-Bond Connectivity (ABC), Randici (RI), Geometric-Arithmetic (GA), Sum-Connectivity (SC), the first and second Zagreb indices ($M_1$ and $M_2$), Schultz second index (SS), Harmonic (H), Hyper-Zagreb (HZ), and the Forgotten index were used on nine drugs, which are linolenic acid, serine, methionine, tyrosine, cystine, succinic acid, N-acetylglucosamine, glutamic Eight basic physicochemical properties were taken into account and the efficacy of the indices was examined by three types of regression straight, logarithmic and quadratic regression. The results of the analysis have shown that there are strong correlations between the chosen topological indices and the physicochemical properties which prove the usefulness of graph-theoretical descriptors in QSPR modeling. The quadratic regression technique was the most predictive of the three models used, and it was better than the linear and logarithmic models. These results indicate the high predictive potential of the topological indices especially in conjunction with non-linear modeling in the interpretation of drug structure-property correlations.

## Introduction

Chemical Graph Theory is a subfield of mathematical chemistry that uses graph theory concepts to study the configurations and structures of chemical compounds. Chemical Graph Theory converts molecules into graphs in which atomic elements become vertices and chemical bonds become edges. The evaluation of physical and chemical properties through topological indices is a core element of this approach, as these mathematical values allow scientists to link the molecular structure to its properties. These widely used mathematical values derived from graphs successfully predict the boiling and melting points, as well as the electronic behavior, infrared spectra, freezing points, viscosity, and density of compounds^1^.

Medical professionals use drugs as chemical substances to identify and treat diseases and prevent and cure diseases. Current healthcare systems base their operations on these therapeutic agents, which help patients manage symptoms, promote healing, and achieve better wellness. Drugs are classified according to their intended functions. Bacterial infections are treated with antibiotics, painkillers relieve discomfort, antipyretics reduce fever, and antihypertensives help manage blood pressure. Drugs, including insulin, serve a dual purpose in the management of diabetes, whereas vaccines serve as preventive measures. Ministerial groups with experience evaluate all pharmaceutical drugs during clinical testing before issuing safety and performance approval. Individuals must follow the guidelines for the appropriate use of medications to obtain the intended therapeutic benefits and reduce side effects.

The human body requires natural compounds such as linolenic acid, serine, methionine, tyrosine, cystine, succinic acid, glutamic acid, glycine, and N-acetylglucosamine to function properly. The healthy fatty acid, linolenic acid, decreases inflammation but benefits the heart. Amino acids, including serine, methionine, and tyrosine, combined with cystine, form proteins that aid in brain performance, body repair, and energy supply. Joint support depends on N-acetylglucosamine, which functions similarly to sugar, helps maintain joint strength; therefore, physicians may prescribe this compound for joint pain relief. The energy production process depends on succinic acid, which has the potential to combat fatigue. These beneficial substances have recently been investigated for the development of new medical treatments across different health domains.

Cheminformatics establishes relationships between QSAR and QSPR models, bioactivities, and chemical compound descriptors using information science and chemical principles, along with mathematical frameworks^2^.

Molecular network evaluation relies on topological indices as key analytical tools to provide structural insights through research-based methods. The use of quantitative structure–property relationships (QSPR) builds valid models that mathematically connect chemical compound structures to their measured properties using statistical, machine learning, or mathematical methods. The connection between molecular descriptors and experimentally observed properties allows QSPR to predict the properties of untested molecules. The widespread application of this method in drug development, materials science, and environmental chemistry optimizes compounds efficiently by bypassing the lengthy and costly experimental methods.

The presentation of chemical molecules via graph structures allows topological indices to function as mathematical numbers that describe the molecular network relationships. Theoretical chemistry depends on such indices to study the connection between the molecular framework and its associated physical and chemical features of the molecular framework. Scientists display molecular compounds using molecular graphs, where vertices (atoms) and edges (bonds) are represented. Chemical formulas allow the creation of graphs that enable researchers to calculate several topological indices^3^. Chemical compounds are represented as molecular graphs in this study using graph theory, and different topological indices are calculated based on vertex degrees^4^. This investigation focuses on the relationship between the efficacy and other properties of the drugs and their molecular structure, as indicated by the topological indices^5^. New indices based on the neighborhood degree sum of nodes are proposed. An algorithm was developed to facilitate the computation of the indices^6^.

By analyzing the molecular structures of drugs used for treatment, topological indices (TIS) are employed to predict their efficacy, toxicity and properties. The paper uses the degree-based TIs and QSPR modeling on drugs, such as glasdegib, palbociclib, and daunorubicin, and emphasizes the use of TIs during the discovery of drugs^7^. The study discusses how topological indices (TIs) and regression modeling can be used to analyze the potential pharmaceutical compounds based on their chemical structures. The present study discusses the application of topological indices (TIs) and linear regression modeling to understand the design of more effective and targeted drug therapy by considering chemical structures of potential pharmaceutical compounds on a degree-based basis. QSPR models rely on degree based TIs to forecast physicochemical properties to guide the construction of more effective and specific drug treatments^8^

Topological indices are an efficient mathematical chemistry methodology that assists chemists and drug scientists in overseeing the chemical and biological activities of chemical compounds or medications^9^. The topological index is derived from molecular structural characteristics, which helps evaluate both the size and shape information of the molecular skeleton without performing any quantum chemical or geometric calculations. Topological indices are fundamental utilities in cheminformatics that can be used to predict molecular characteristics, biological activities, and physicochemical properties using structural data alone.

Topological indices emerged as an important analytical tool in 1947^10^ when Wiener introduced them to molecular chemistry^11^. This study evaluated various topological index categories based on distance, resistance, and eccentricity information^12^. This study examines the possible uses based on the mathematical characteristics of the normalized Laplacian matrix of this particular graphene allotrope^13^. This study focuses on the spectral analysis of three random-walk invariants on a particular type of network, namely rounded networks with two n-pentagons^14^. Using distance-based methods, this study examined topological polynomials related to zero-divisor graphs^15^. This study aims to identify highly connected regions within the molecular graph and relate them to the compound’s physical properties^16^. These findings present encouraging opportunities for chemical and materials engineering applications, particularly in chemical industry research^17^. The investigation emphasizes the mathematical characteristics and computations associated with these indices, which are utilized to forecast molecular properties in chemical graph theory^18^. Finding connected graphs that maximize this particular topological index is the main goal of the study^19^–^21^. The use of minimum Zagreb eccentricity indices in two-mode networks is investigated in this study, with particular reference to benzenoid hydrocarbons and boiling points^22^. In many disciplines, including chemistry, electronics, business and economics, medicine, and social sciences, the value of various topological indices is unavoidable.^23^. Recent developments in graph-structured learning have shown encouraging outcomes^24^. It examines random walks on an octagonal cell network, a particular type of network^25^. The selected carbon nanotube were analyzed in the study using topological indices and chemical graph theory^26^ to^27^.This study focuses on the mathematical features and spectral properties of a particular type of graph, known as the K4 chain graph^28^. Some of the topological indices that are questioned are the Atom-Bond Connectivity (ABC) index, Randic (RI), Geometric-Arithmetic (GA), Sum connectivity (SC), first and second Zagreb (M1 and M2) index, Schultz second index (SS), Hyper Zagreb (HZ) index, Harmonic (H) and Forgotten (F) index. The molecular formulas of various drugs produce numerical values that these indices derive and help analyze the structural qualities of drugs^29^.

## Preliminaries

### Definition 1

In graph theory, a graph *G* is defined as a pair of things in order. $G = (V, E)$, where *V* is a non-empty finite subset of elements known as vertices and *E* is a collection of unordered pairs of different elements of *V*, called edges. A graph is a simple mathematical structure, in basic terms, consists of a set of points, called vertices, and a set of lines, called edges, which interconnect some pairs of points.

### Definition 2

The vertex degree signifies the number of edges incident on a vertex. By the symbol $d_v (G)$ or $d_v$ the degree of a vertex is expressed.

### Definition 3

In a graph, two vertices that share an adjacent edge are adjacent vertices. Two edges with common nodes are adjacent.

### Definition 4

The neighbourhood of a vertex *v* in a graph, denoted by *N*(*v*), is the set of all vertices that are adjacent to vertex *v*

$$N(v) = \{ u \in V(G) \mid uv \in E(G) \}$$

### Definition 5

In 1975, Milan Randić’s seminal paper “On Characterization of Molecular Branching” proposed the first degree-based topological index^30^. This index, which came to be known as the Randić index, was defined as:

1 $$\begin{aligned} R(G)= \sum _{uv \in E(G)} \frac{1}{\sqrt{d_u(G) d_v(G)}} \end{aligned}$$

### Definition 6

Harmonic index was first proposed by Fajtlowicz as:

2 $$\begin{aligned} H(G)= \sum _{uv \in E(G)} \frac{2}{d_u(G) + d_v(G)} \end{aligned}$$

### Definition 7

These terms provide quantitative measurements of molecular branching because they increase in proportion to the branching of the carbon atom skeleton. In a review paper published ten years later, Balaban^31^ mentioned $M_1$ and $M_2$ as topological indices and called them the “Zagreb group indices.”

3 $$\begin{aligned} & M_1(G) = \sum _{v \in V(G)} d_v(G)^2 \end{aligned}$$

4 $$\begin{aligned} M_2(G) = \sum _{uv \in E(G)} d_u(G) d_v(G) \end{aligned}$$

### Definition 8

The expression for Forgotten Topological Index^32^ is

5 $$\begin{aligned} F(G) = \sum _{v \in V(G)} d_v(G)^3 \end{aligned}$$

### Definition 9

The “atom-bond connectivity index”^33^, commonly abbreviated as ABC, is defined as

6 $$\begin{aligned} ABC(G) = \sum _{uv \in E(G)} \sqrt{\frac{d_u(G) + d_v(G) - 2}{d_u(G) d_v(G)}} \end{aligned}$$

### Definition 10

Schultz’s Second Index is defined for a molecular graph G as follows:

7 $$\begin{aligned} SS(G) = \sum _{uv \in E(G)} \frac{1}{(d_u(G) + d_v(G))^2} \end{aligned}$$

### Definition 11

The difference between the geometric and arithmetic means is the basis for another newly created vertex-degree-based topological index, called the geometric-arithmetic (GA) index, which is defined as

8 $$\begin{aligned} GA(G) = \sum _{uv \in E(G)} \frac{2\sqrt{d_u(G) d_v(G)}}{d_u(G) + d_v(G)} \end{aligned}$$

### Definition 12

Zhou and Trinajstić introduced a more recent topological index called the “sum-connectivity index.”^34^.

9 $$\begin{aligned} SCI(G) = \sum _{uv \in E(G)} \frac{1}{\sqrt{d_u(G) + d_v(G)}} \end{aligned}$$

### Definition 13

The hyper-Zagreb index is defined for a molecular graph G as follows:

10 $$\begin{aligned} HZ(G) = \sum _{uv \in E(G)} \left( d_u(G) + d_v(G) \right) ^2 \end{aligned}$$

## Methodology and analysis

**Fig. 1:**
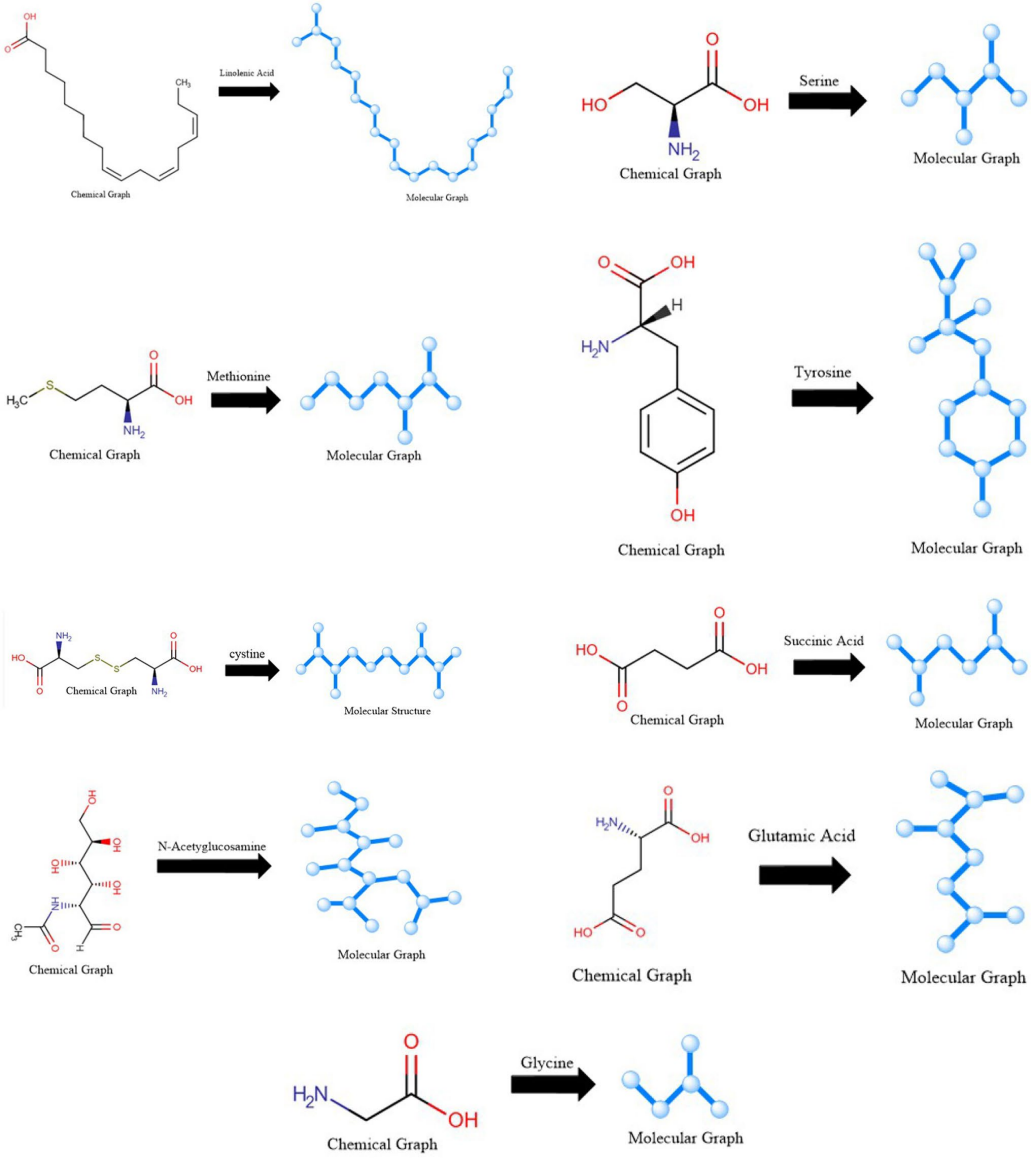
Chemical structures of the drugs.

In this section, ten topological indices are considered to model eight physical properties commonly found in nine drugs: linolenic acid, serine, methionine, tyrosine, cystine, succinic acid, N-acetylglucosamine, glutamic acid, and glycine. These drugs were used to compute the corresponding values for each property. The chemical structures of these ten drugs are shown in Fig. 1.

Edge distribution analysis was used to investigate the structural connectivity patterns of nine biochemical compounds. For clarity, the edge distribution of each compound is displayed in nine separate tables. Table 1 displays the edge distribution of linolenic acid, whereas Tables 2, 3, 4 and 5 display the distributions of serine, methionine, tyrosine, and cysteine, respectively. Tables 6, 7, 8 and 9 display the corresponding data for glycine, glutamic acid, succinic acid, and N-acetylgl. This analysis provides valuable information on the structural features and networks of interactions of these compounds, advancing our understanding of their chemical composition and potential biological roles.

**Table 1:** Partitioning of linolenic acid based on its degree.

(1,2)	(1,3)	(1,4)	(2,2)	(2,3)	(2,4)	(3,3)	(3,4)
1	2	0	15	1	0	0	0

**Table 2:** Partitioning of serine based on its degree.

(1,2)	(1,3)	(1,4)	(2,2)	(2,3)	(2,4)	(3,3)	(3,4)
1	3	0	0	1	0	1	0

**Table 3:** Partitioning of methionine based on its degree.

(1,2)	(1,3)	(1,4)	(2,2)	(2,3)	(2,4)	(3,3)	(3,4)
1	3	0	2	1	0	1	0

**Table 4:** Partitioning of tyrosine based on its degree.

(1,2)	(1,3)	(1,4)	(2,2)	(2,3)	(2,4)	(3,3)	(3,4)
0	3	2	2	5	1	0	1

**Table 5:** Partitioning of cystine based on its degree.

(1,2)	(1,3)	(1,4)	(2,2)	(2,3)	(2,4)	(3,3)	(3,4)
0	6	0	3	2	0	2	0

**Table 6:** Partitioning of succine acid based on its degree.

(1,2)	(1,3)	(1,4)	(2,2)	(2,3)	(2,4)	(3,3)	(3,4)
0	4	0	1	2	0	0	0

**Table 7:** Partitioning of N-acctylglucosamine based on its degree.

(1,2)	(1,3)	(1,4)	(2,2)	(2,3)	(2,4)	(3,3)	(3,4)
1	7	0	0	3	0	4	0

**Table 8:** Partitioning of glutamic acid based on its degree.

(1,2)	(1,3)	(1,4)	(2,2)	(2,3)	(2,4)	(3,3)	(3,4)
0	5	0	1	2	0	1	0

**Table 9:** Partitioning of glycine based on its degree.

(1,2)	(1,3)	(1,4)	(2,2)	(2,3)	(2,4)	(3,3)	(3,4)
1	2	0	0	1	0	0	0

### Physico-chemical properties of drugs

The physical and chemical characteristics of these medications are shown in Table 10 namely, boiling points(BP), molar volumes (MV), molar refraction (MR), molecular weight (MW), heavy atom count (HAV), complexity (CO), enthalpy of vaporization (EV), and melting point (MP). Table 11 lists the topological characteristics of these drugs. Regression analysis was performed using SPSS. All the drugs have been taken from the website https://go.drugbank.com.

**Table 10:** Various drugs along with their physical and chemical properties.

Sr.No	Drugs	${ {BP}} (^0 C)$	${ {MV}} ({ {cm}}^3/{ {mol}})$	${ {MR}} ({ {cm}}^3/{ {mol}})$	${ {MW}} ({ {g/mol}})$	HAC	CO	EV (${ {KJ/mol}}$)	${ {MP}} (^0 C)$
1	Linolenic acid	496	256.9	89.97	278.44	22	287	93.7	164.33
2	Serine	304.87	91.2	21.88	105.09	10	102	72.5	139.6
3	Methionine	327.23	105.4	37.35	149.21	14	128	78.0	135.72
4	Tyrosine	457.27	164.2	46.62	181.19	16	174	90.2	284.54
5	Cystine	636.49	177.5	53.89	240.29	18	195	84.6	410.25
6	Succinic acid	309.01	106.2	21.93	118.09	10	82	73.9	182.39
7	N-Acetylglucosamine	650.56	227.3	49.13	221.21	21	241	96.4	297.76
8	Glutamic acid	403.96	127.6	30.33	147.13	13	115	76.5	261.92
9	Glycine	190.49	64.1	16.02	75.07	7	55	62.8	82.51

**Table 11:** Drugs with their computed topological values.

Sr. No	Drugs	M$_{1}$	M$_{2}$	H	F	SS	ABC	RI	SC	GA	HZ
1	Linolenic acid	76.0000	74.0000	9.5667	158.0000	18.6440	13.6538	9.7701	9.5246	18.6547	306.0000
2	Serine	26.0000	26.0000	2.9000	66.0000	5.7348	4.5304	3.1807	2.9328	5.5207	118.0000
3	Methionine	34.0000	34.0000	3.9000	82.0000	7.7348	5.9446	4.1807	3.9328	7.5207	150.0000
4	Tyrosine	68.0000	75.0000	5.9190	190.0000	14.3282	10.4839	6.4155	6.4167	13.0296	340.0000
5	Cystine	58.0000	60.0000	5.9667	146.0000	12.8365	9.7678	6.4473	6.2109	12.1557	266.0000
6	Succinic acid	30.0000	28.0000	3.3000	74.0000	6.6550	5.3873	3.6259	3.3944	6.4237	130.0000
7	N-Acetylglucosamine	70.0000	77.0000	6.7000	186.0000	15.0640	11.2106	7.3066	7.0520	13.9444	340.0000
8	Glutamic acid	40.0000	40.0000	4.1333	102.0000	8.7458	6.8705	4.5366	4.3027	8.2897	182.0000
9	Glycine	16.0000	14.0000	2.0667	38.0000	3.6440	3.0472	2.2701	2.0246	3.6547	66.0000

Topological indices like the Atom-Bond Connectivity (ABC), Randic (RI), Geometric-Arithmetic (GA), Sum-Connectivity (SC), Zagreb ($M_1$ and $M_2$), Schultz second (SS), Hyper-Zagreb (HZ), Harmonic (H) and Forgotten (F) indices are important in QSPR because of their ability to transform molecular structures into numerical indices to describe branching, connectivity and complexity. These indices have extensively been used to predict physicochemical and biological characteristics, including stability, solubility, boiling point, and activity thereby making them useful in modelling the relationship between structure and properties and supporting effective drug design and testing. The results of each regression model are presented in separate tables to facilitate comparison. Table 12 displays the results of the linear regression model, Table 13 displays the results of the quadratic regression model, and Table 14 displays the results of the logarithmic regression model.

In Table 12, we have shown that which topological index is the best predictor on the basis of the highest value of $R^{2}$. In this study, the “best predictor” refers to the descriptors yielding the highest coefficient of determination ($R^{2}$), which indicates the strongest explanatory power of the regression model. This approach is commonly used in QSPR studies^35^. Best predictor is predicting the physicochemical properties on the basis of highest value of $R^{2}$.

**Table 12:** Outcomes of Linear Regression Model.

Property	Predictors	$R^{2}$	Best Predictor
BP	M$_{1}$	0.735	HZ
	M$_{2}$	0.756	
	F	0.756	
	HZ	0.760	
MV	M$_{1}$	0.914	RI
	M$_{2}$	0.859	
	H	0.952	
	F	0.759	
	SS	0.950	
	ABC	0.957	
	RI	0.967	
	SC	0.960	
	GA	0.956	
*MR*	M$_{1}$	0.766	H
	H	0.955	
	SS	0.866	
	ABC	0.864	
	RI	0.934	
	SC	0.928	
	GA	0.915	
MW	–	–	–
HAC	M$_{1}$	0.903	ABC
	M$_{2}$	0.868	
	H	0.901	
	F	0.780	
	SS	0.921	
	ABC	0.927	
	RI	0.923	
	SC	0.917	
	GA	0.916	
	HZ	0.824	
CO	M$_{1}$	0.893	RI
	M$_{2}$	0.840	
	H	0.954	
	F	0.728	
	SS	0.939	
	ABC	0.939	
	RI	0.962	
	SC	0.956	
	GA	0.950	
	HZ	0.783	
EV	M$_{1}$	0.949	M$_{2}$
	M$_{2}$	0.955	
	H	0.820	
	F	0.915	
	SS	0.916	
	ABC	0.915	
	RI	0.856	
	SC	0.863	
	GA	0.878	
	HZ	0.938	
MP	–	–	–

**Table 13:** Outcomes of Quadratic Regression Model.

Property	Predictors	$R^{2}$	Best Predictor
BP	M$_{1}$	0.809	H
	M$_{2}$	0.808	
	H	0.849	
	F	0.816	
	SS	0.819	
	ABC	0.818	
	RI	0.844	
	SC	0.835	
	GA	0.828	
	HZ	0.812	
MV	M$_{1}$	0.923	RI
	M$_{2}$	0.859	
	H	0.961	
	F	0.789	
	SS	0.954	
	ABC	0.962	
	RI	0.968	
	SC	0.961	
	GA	0.956	
	HZ	0.817	
MR	M$_{1}$	0.789	H
	H	0.965	
	SS	0.908	
	ABC	0.912	
	RI	0.955	
	SC	0.952	
	GA	0.944	
MW	M$_{1}$	0.861	H
	M$_{2}$	0.826	
	H	0.950	
	F	0.808	
	SS	0.908	
	ABC	0.918	
	RI	0.948	
	SC	0.938	
	GA	0.928	
	HZ	0.815	
HAC	M$_{1}$	0.906	H
	M$_{2}$	0.883	
	H	0.957	
	F	0.841	
	SS	0.930	
	ABC	0.932	
	RI	0.956	
	SC	0.946	
	GA	0.939	
	HZ	0.859	
CO	M$_{1}$	0.904	RI
	M$_{2}$	0.840	
	H	0.960	
	F	0.759	
	SS	0.944	
	ABC	0.948	
	RI	0.963	
	SC	0.956	
	GA	0.951	
	HZ	0.792	
EV	M$_{1}$	0.954	M$_{2}$
	M$_{2}$	0.958	
	H	0.940	
	F	0.929	
	SS	0.947	
	ABC	0.941	
	RI	0.940	
	SC	0.941	
	GA	0.943	
	HZ	0.945	
MP	H	0.760	H
	SS	0.743	
	ABC	0.737	
	RI	0.758	
	SC	0.758	
	GA	0.756

**Table 14:** Outcomes of Logarithmic Regression Model.

Property	Predictors	$R^{2}$	Best Predictors
BP	M$_{1}$	0.777	F, HZ
	M$_{2}$	0.787	
	H	0.709	
	F	0.791	
	SS	0.752	
	ABC	0.761	
	RI	0.733	
	SC	0.730	
	GA	0.733	
	HZ	0.791	
MV	M$_{1}$	0.852	H
	M$_{2}$	0.812	
	H	0.933	
	F	0.760	
	SS	0.887	
	ABC	0.893	
	RI	0.931	
	SC	0.919	
	GA	0.906	
	HZ	0.786	
MR	M$_{1}$	0.700	H
	H	0.868	
	SS	0.769	
	ABC	0.768	
	RI	0.845	
	SC	0.831	
	GA	0.812	
MW	M$_{1}$	0.8647	H
	M$_{2}$	0.804	
	H	0.942	
	F	0.744	
	SS	0.888	
	ABC	0.894	
	RI	0.937	
	SC	0.925	
	GA	0.912	
	HZ	0.774	
HAC	M$_{1}$	0.891	RI
	M$_{2}$	0.869	
	H	0.948	
	F	0.814	
	SS	0.918	
	ABC	0.918	
	RI	0.949	
	SC	0.939	
	GA	0.931	
	HZ	0.842	
CO	M$_{1}$	0.830	RI
	M$_{2}$	0.797	
	H	0.928	
	F	0.731	
	SS	0.871	
	ABC	0.870	
	RI	0.921	
	SC	0.908	
	GA	0.894	
	HZ	0.764	
EV	M$_{1}$	0.947	M$_{1}$, M$_{2}$
	M$_{2}$	0.947	
	H	0.913	
	F	0.924	
	SS	0.943	
	ABC	0.937	
	RI	0.924	
	SC	0.927	
	GA	0.933	
	HZ	0.937	
MP	–	–	–

## Regression models and statistical analysis

Three regression models were used to determine which model provided the most accurate estimation of the connection between the physicochemical characteristics and topological indices.

11 $$\begin{aligned} & P = \alpha + \beta (T) \end{aligned}$$

12 $$\begin{aligned} & P = \alpha + \beta (T) + \gamma (T)^2 \end{aligned}$$

13 $$\begin{aligned} & P = \alpha + \beta (lnT) \end{aligned}$$

where P represents a physical attribute, T represents the topological index, and $\alpha$, $\beta$ and $\gamma$ are constants.

An analysis of the linear and quadratic regression models was performed at two points using the data in Table 10 with computed values in Table 11.

In linear regression analysis, $\alpha$ is a constant, $\beta$ is the regression coefficient, the statistical F is (F-value), the correlation coefficient (R), the P-value (p), the coefficient of determination $(R^{2})$, and the standardized residuals (S). In quadratic regression, $\alpha$ is a constant, $\beta$ is the topological index coefficient, and $\gamma$ is the coefficient of the index square. In logarithmic regression, the regression coefficient is denoted by $\alpha$, and $\beta$ is a constant. To seek model fit and discover outliers, standardized residuals (the residuals divided by their standard deviations) were used. N is usually a measure of the number of data points assumed in regression analysis. If the P-value $\le 0.05$, then there is a statistically significant relationship between the predictor variables and the response variable. If the F-value $>2.5$, then the model improves on the mere application without any predictors. R, that is, correlation coefficient ought to be as close to 1 if the relationship is positive and as close to negative 1 if the association is negative; values that are above 0.8 are tentatively treated as an indication of a strong correlation. Table 15, Table 16, and Table 17 show the regression equations and statistical parameters for the topological indices.

The indices proposed by the linear regression model can be used to forecast HZ, RI, H, ABC, and $M_2$, as shown in Table 12. Table 14 demonstrates that the suggested indices are used with the logarithmic regression model, H, RI, HZ, and $M_2$. Additionally, the suggested indices are used in the quadratic regression models F, HZ, H, RI, $M_1$, and $M_2$ in Table 13.

**Table 15:** Statistical calculation of best predictors of linear regression models.

Regression Equation	N	$\alpha$	$\beta$	p	F	R	$R^{2}$	S$_{\hbox {res}}$
1.309(HZ)+143.405	9	143.405	1.309	0.002	22.167	0.872	0.76	81.619
26.866(RI)+4.223	9	4.223	26.866	0.001	202.938	0.983	0.967	12.634
9.531(H)-6.305	9	− 6.305	9.531	0.001	148.595	0.977	0.955	5.148
1.4(ABC)+3.525	9	3.525	1.4	0.001	88.803	0.963	0.927	1.488
31.820(RI)-15.543	9	− 15.543	31.82	0.001	178.806	0.981	0.962	15.942
0.447($M_2$)+59.715	9	59.715	0.447	0.001	149.224	0.977	0.955	2.504

**Table 16:** Statistical calculation of best predictors of quadratic regression models.

Regression Equation	N	$\alpha$	$\beta$	p	F	R	$R^{2}$	S$_{\hbox {res}}$	$\gamma$
-231.762+218.419(H)-14.634$(H)^{2}$	9	231.762	218.419	0.007	12.926	0.901	0.968	78.103	− 14.634
-9.117+32.180(RI)-0.448$(RI)^{2}$	9	-9.117	32.18	0.001	91.011	0.984	0.968	13.35	− 0.448
− 4.710+4.920(H)+0.403$(H)^{2}$	9	− 4.71	4.92	0.001	82.097	0.982	0.965	4.923	0.403
− 23.623+50.708(H)− 2$(H)^{2}$	9	− 23.623	50.708	0.001	57.525	0.975	0.95	17.317	− 2
− 1.807+4.607(H)-0.219$(H)^{2}$	9	-1.807	4.607	0.001	66.432	0.978	0.957	1.236	-0.219
− 24.019+35.197(RI)-0.285$(RI)^{2}$	9	− 24.019	35.197	0.001	77.508	0.981	0.963	17.125	− 0.285
− 56.562+0.614($M_2$)− 0.002$(M_2)^{2}$	9	− 56.562	0.614	0.001	69.051	0.979	0.958	2.607	− 0.002
− 261.572+180.608(H)− 14.131$(H)^{2}$	9	− 261.572	180.608	0.001	9.515	0.872	0.76	58.308	− 14.13

**Table 17:** Statistical calculation of best predictors of logarithm regression models.

Regression Equation	N	$\alpha$	$\beta$	p	F	R	$R^{2}$	S$_{\hbox {res}}$
− 756.851+254.008(*lnF*)	9	− 756.851	254.008	0.001	26.465	0.889	0.791	76.13
− 860.308+245.039(*lnHZ*)	9	− 860.308	245.039	0.001	26.465	0.889	0.791	76.198
− 50.541+131.605(*lnH*)	9	− 50.541	131.605	0.001	96.717	0.966	0.933	17.975
− 26.008+44.554(*lnH*)	9	− 26.008	44.554	0.001	46.178	0.932	0.868	8.807
− 37.946+137.681(*lnH*)	9	− 37.946	137.681	0.001	113.991	0.971	0.942	17.321
− 2.699+10.937(lnRI)	9	− 2.699	10.937	0.001	129.462	0.974	0.949	1.247
− 100.370+160.748(lnRI)	9	− 100.37	160.748	0.001	81.671	0.96	0.921	23.076
5.68+20.219($lnM_1$)	9	5.68	20.219	0.001	125.557	0.973	0.947	2.718
13.175+18.207($lnM_2$)	9	13.175	18.207	0.001	125.733	0.973	0.947	2.716

**Fig. 2:**
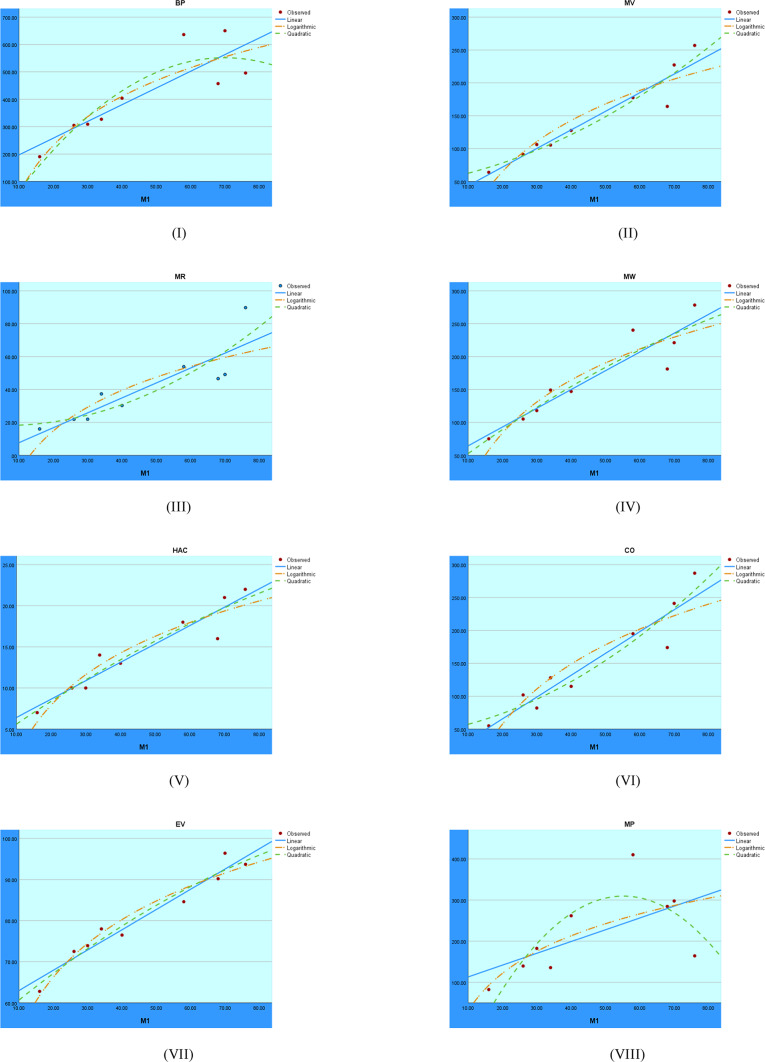
Visual representations of the regression analysis conducted on the $M_1$ index.

The regression analysis of the First Zagreb index using linear, quadratic, and logarithmic models is shown graphically in Fig. 2. These graphs show the accuracy with which each model depicts the connection between the target variable and the First Zagreb index. Comparing the trends makes it clear which model fits the data best, which helps assess the predictability of the structural properties.

After analyzing the indices in the quadratic regression model in Table 13, the results show that defining $M_1$ makes it possible to create the following models to predict BP, MV, MR, MW, HAC, CO, and EV:

14 $$\begin{aligned} BP&= -0.135(M_1)^{2}+18.913(M_1)-109.343 \end{aligned}$$

15 $$\begin{aligned} MV&= 0.020(M_1)^{2}+0.972(M_1)-51.038 \end{aligned}$$

16 $$\begin{aligned} MR&= 0.011(M_1)^{2}-0.132(M_1)-18.555\end{aligned}$$

17 $$\begin{aligned} MW&= -0.012(M_1)^{2}+4.003(M_1)-13.917 \end{aligned}$$

18 $$\begin{aligned} HAC&= -0.001(M_1)^{2}+0.302(M_1)+2.669 \end{aligned}$$

19 $$\begin{aligned} CO&= 0.0026(M_1)^{2}+0.878(M_1)+46.001 \end{aligned}$$

20 $$\begin{aligned} EV&= -0.002(M_1)^{2}+0.720(M_1)+53.731 \end{aligned}$$

The models below predict BP, MV, MW, HAC, CO, and EV using $M_2$ in quadratic regression.

21 $$\begin{aligned} BP&= -0.099(M_2)^{2}+15.151(M_2)-26.703 \end{aligned}$$

22 $$\begin{aligned} MV&= 0.001(M_2)^{2}+2.383(M_2)+30.680 \end{aligned}$$

23 $$\begin{aligned} MW&= -0.034(M_2)^{2}+5.816(M_2)-12.297 \end{aligned}$$

24 $$\begin{aligned} HAC&= -0.002(M_2)^{2}+0.367(M_2)+1.955 \end{aligned}$$

25 $$\begin{aligned} CO&= -0.001(M_2)^{2}+3.017(M_2)+12.862 \end{aligned}$$

26 $$\begin{aligned} EV&= -0.002(M_2)^{2}+0.614(M_2)+56.562 \end{aligned}$$

The models below predict BP, MV, MR, MW, HAC, CO, EV, and MP using H in a quadratic regression.

27 $$\begin{aligned} BP&= -14.634(H)^{2}+218.419(H)-23.762 \end{aligned}$$

28 $$\begin{aligned} MV&= -1.103(H)^{2}+39.751(H)-17.412 \end{aligned}$$

29 $$\begin{aligned} MR&= 0.403(H)^{2}+4.920(H)+4.710 \end{aligned}$$

30 $$\begin{aligned} MW&= -2.000(H)^{2}+50.708(H)-12.297 \end{aligned}$$

31 $$\begin{aligned} HAC&= -0.219(H)^{2}+4.607(H)-1.807 \end{aligned}$$

32 $$\begin{aligned} CO&= -1.084(H)^{2}+44.648(H)-35.627 \end{aligned}$$

33 $$\begin{aligned} EV&= -0.689(H)^{2}+12.193(H)+40.852 \end{aligned}$$

34 $$\begin{aligned} MP&= -14.131(H)^{2}+180.608(H)-261.572 \end{aligned}$$

The models below predict BP, MV, MW, HAC, CO, and EV using F in a quadratic regression.

35 $$\begin{aligned} BP&= -0.018(F)^{2}+6.659(F)+-64.373 \end{aligned}$$

36 $$\begin{aligned} MV&= -0.005(F)^{2}+2.255(F)-29.989 \end{aligned}$$

37 $$\begin{aligned} MW&= -0.011(F)^{2}+3.547(F)-69.629 \end{aligned}$$

38 $$\begin{aligned} HAC&= -0.001(F)^{2}+0.224(F)-1.687 \end{aligned}$$

39 $$\begin{aligned} CO&= -0.006(F)^{2}+2.689(F)-55.445 \end{aligned}$$

40 $$\begin{aligned} EV&= -0.001(F)^{2}+0.337(F)+51.918 \end{aligned}$$

The models below predict BP, MV,MR, MW, HAC, CO, EV, and MP using SS in a quadratic regression.

41 $$\begin{aligned} BP&= -2.911(SS)^{2}+89.697(SS)-132.282 \end{aligned}$$

42 $$\begin{aligned} MV&= 0.180(SS)^{2}+8.581(SS)+34.288 \end{aligned}$$

43 $$\begin{aligned} MR&= 0.227(SS)^{2}-0.798(SS)+19.555 \end{aligned}$$

44 $$\begin{aligned} MW&= -0.166(SS)^{2}+16.413(SS)+19.726 \end{aligned}$$

45 $$\begin{aligned} HAC&= -0.024(SS)^{2}+1.508(SS)+2.006 \end{aligned}$$

46 $$\begin{aligned} CO&= 0.274(SS)^{2}+8.768(SS)+26.644 \end{aligned}$$

47 $$\begin{aligned} EV&= -0.096(SS)^{2}+4.222(SS)+49.632 \end{aligned}$$

48 $$\begin{aligned} MP&= -3.431(SS)^{2}+86.680(SS)-235.166 \end{aligned}$$

The models below predict BP, MV,MR, MW, HAC, CO, EV, and MP using ABC in a quadratic regression.

49 $$\begin{aligned} BP&= -5.349(ABC)^{2}+125.241(ABC)-175.445 \end{aligned}$$

50 $$\begin{aligned} MV&= 0.466(ABC)^{2}+10.161(ABC)+32.549 \end{aligned}$$

51 $$\begin{aligned} MR&= 0.487(ABC)^{2}-2.096(ABC)+21.667 \end{aligned}$$

52 $$\begin{aligned} MW&= -0.185(ABC)^{2}+21.282(ABC)+14.331 \end{aligned}$$

53 $$\begin{aligned} HAC&= -0.035(ABC)^{2}+1.987(ABC)+1.497 \end{aligned}$$

54 $$\begin{aligned} CO&= 0.717(ABC)^{2}+9.142(ABC)+28.689 \end{aligned}$$

55 $$\begin{aligned} EV&= -0.174(ABC)^{2}+5.863(ABC)+47.480 \end{aligned}$$

56 $$\begin{aligned} MP&=-6.635(ABC)^{2}+125.919(ABC)-288.585 \end{aligned}$$

The models below predict BP, MV,MR, MW, HAC, CO, EV, and MP using RI in a quadratic regression.

57 $$\begin{aligned} BP&= -13.354(RI)^{2}+210.292(RI)-253.555 \end{aligned}$$

58 $$\begin{aligned} MV&= -0.448(RI)^{2}+32.180(RI)-9.117 \end{aligned}$$

59 $$\begin{aligned} MR&= 0.591(RI)^{2}+2.255(RI)+9.225 \end{aligned}$$

60 $$\begin{aligned} MW&= -1.439(RI)^{2}+44.544(RI)-20.177 \end{aligned}$$

61 $$\begin{aligned} HAC&= -0.170(RI)^{2}+4.110(RI)-1.596 \end{aligned}$$

62 $$\begin{aligned} CO&= -0.285(RI)^{2}+35.197(RI)-24.019 \end{aligned}$$

63 $$\begin{aligned} EV&= -0.589(RI)^{2}+11.306(RI)+40.507 \end{aligned}$$

64 $$\begin{aligned} MP&= -13.887(RI)^{2}+184.879(RI)-303.007 \end{aligned}$$

The models below predict BP, MV,MR, MW, HAC, CO, EV, and MP using SC with quadratic regression.

65 $$\begin{aligned} BP&= -13.263(SC)^{2}+202.009(SC)-197.996 \end{aligned}$$

66 $$\begin{aligned} MV&= -0.332(SC)^{2}+30.386(SC)+2.377 \end{aligned}$$

67 $$\begin{aligned} MR&= 0.644(SC)^{2}+1.873(SC)+11.312 \end{aligned}$$

68 $$\begin{aligned} MW&= -1.330(SC)^{2}+42.303(SC)-5.682 \end{aligned}$$

69 $$\begin{aligned} HAC&= -0.162(SC)^{2}+3.912(SC)-0.325 \end{aligned}$$

70 $$\begin{aligned} CO&= -0.133(SC)^{2}+33.028(SC)-10.710 \end{aligned}$$

71 $$\begin{aligned} EV&= -0.568(SC)^{2}+10.758(SC)+43.791 \end{aligned}$$

72 $$\begin{aligned} MP&= -13.890(SC)^{2}+177.985(SC)-258.255 \end{aligned}$$

The models below predict BP, MV,MR, MW, HAC, CO, EV, and MP using GA in a quadratic regression.

73 $$\begin{aligned} BP&= -3.253(GA)^{2}+96.825(GA)-153.144 \end{aligned}$$

74 $$\begin{aligned} MV&= -0.023(GA)^{2}+13.603(GA)+14.581 \end{aligned}$$

75 $$\begin{aligned} MR&= 0.181(GA)^{2}+0.546(GA)+13.887 \end{aligned}$$

76 $$\begin{aligned} MW&= -0.289(GA)^{2}+19.716(GA)+7.439 \end{aligned}$$

77 $$\begin{aligned} HAC&= -0.037(GA)^{2}+1.828(GA)+0.822 \end{aligned}$$

78 $$\begin{aligned} CO&= 0.037(GA)^{2}+14.703(GA)+3.092 \end{aligned}$$

79 $$\begin{aligned} EV&= -0.132(GA)^{2}+5.037(GA)+46.730 \end{aligned}$$

80 $$\begin{aligned} MP&= -3.478(GA)^{2}+86.524(GA)-226.130 \end{aligned}$$

The models below predict BP, MV, MW, HAC, CO, and EV using HZ in a quadratic regression.

81 $$\begin{aligned} BP&= -0.005(HZ)^{2}+3.493(HZ)-42.636 \end{aligned}$$

82 $$\begin{aligned} MV&= -0.001(HZ)^{2}+0.915(HZ)-1.752 \end{aligned}$$

83 $$\begin{aligned} MW&= -0.003(HZ)^{2}+1.680(HZ)-45.017 \end{aligned}$$

84 $$\begin{aligned} HAC&= 0.000(HZ)^{2}+0.104(HZ)-0.012 \end{aligned}$$

85 $$\begin{aligned} CO&= -0.001(HZ)^{2}+1.113(HZ)-23.932 \end{aligned}$$

86 $$\begin{aligned} EV&= 0.000(HZ)^{2}+0.159(HZ)+54.431 \end{aligned}$$

**Fig. 3:**
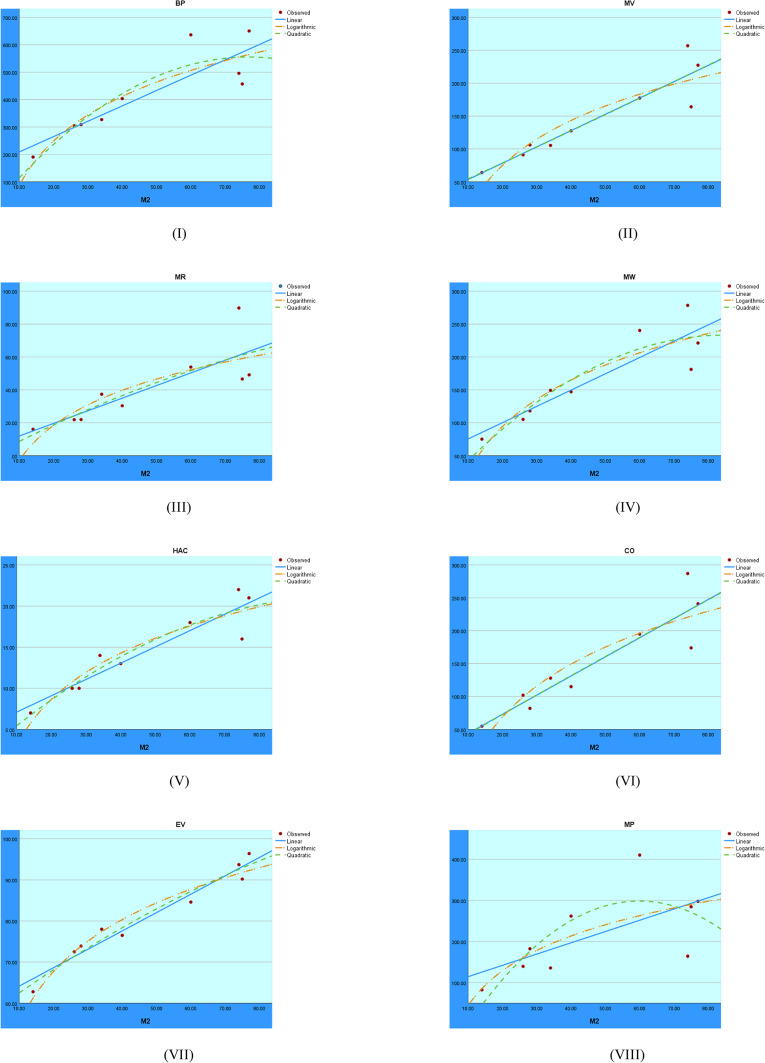
Visual representations of the regression analysis conducted on the $M_2$ index.

The analysis of the Second Zagreb index using linear, quadratic, and logarithmic models is shown graphically in Fig. 3. These figures make it evident how each model describes the connection between the dependent variable and the Second Zagreb index. It is possible to determine which model has a higher correlation and superior predictive accuracy for the Second Zagreb index by comparing these regression curves.

**Fig. 4:**
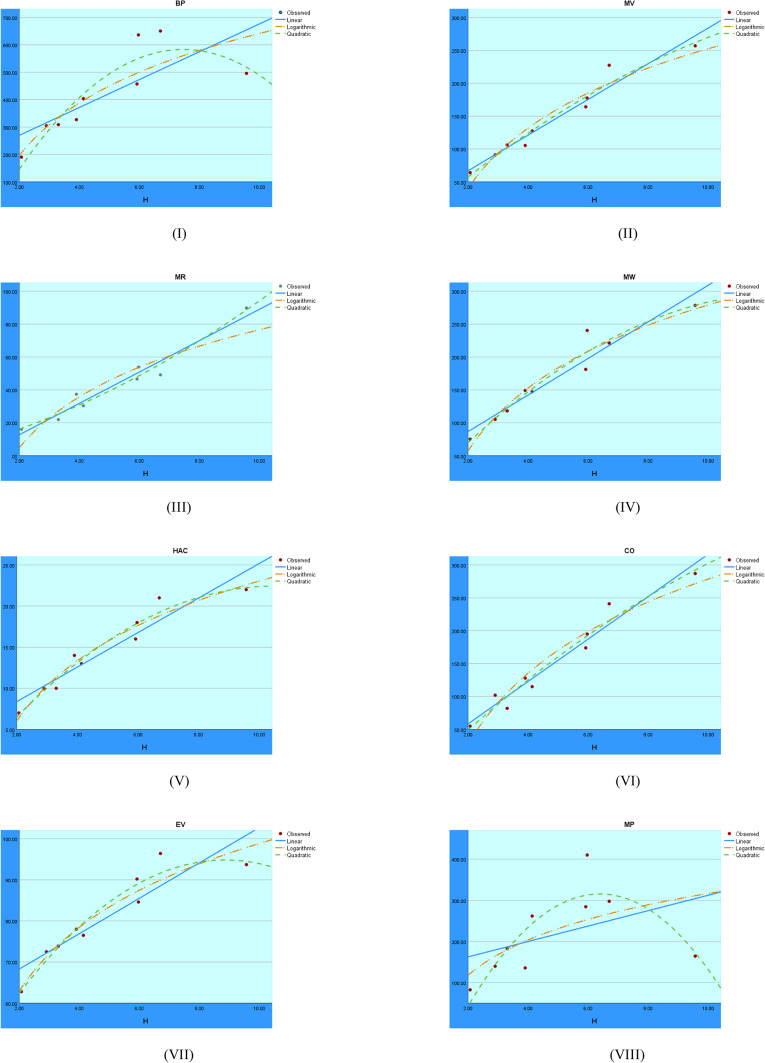
Visual representations of the regression analysis conducted on the H index.

A visual representation of the logarithmic, quadratic, and linear regression models applied to the H index is shown in Fig. 4. The comparison shows how well each model fits the data and the strength of the relationship between the target variable and the H index.

**Fig. 5:**
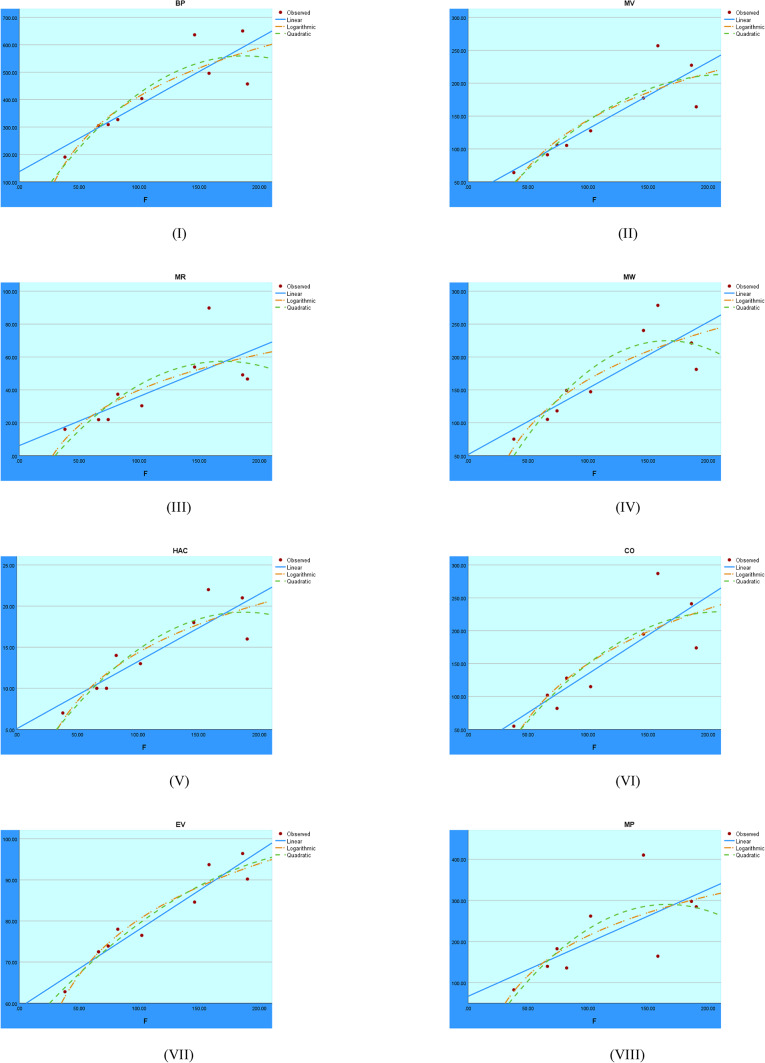
Visual representations of the regression analysis conducted on the F index.

The regression analysis for the F index using logarithmic, quadratic, and linear models is shown in Fig. 5. The curves make it easier to see how well each model captures the relationship between the dependent variable and F index.

**Fig. 6:**
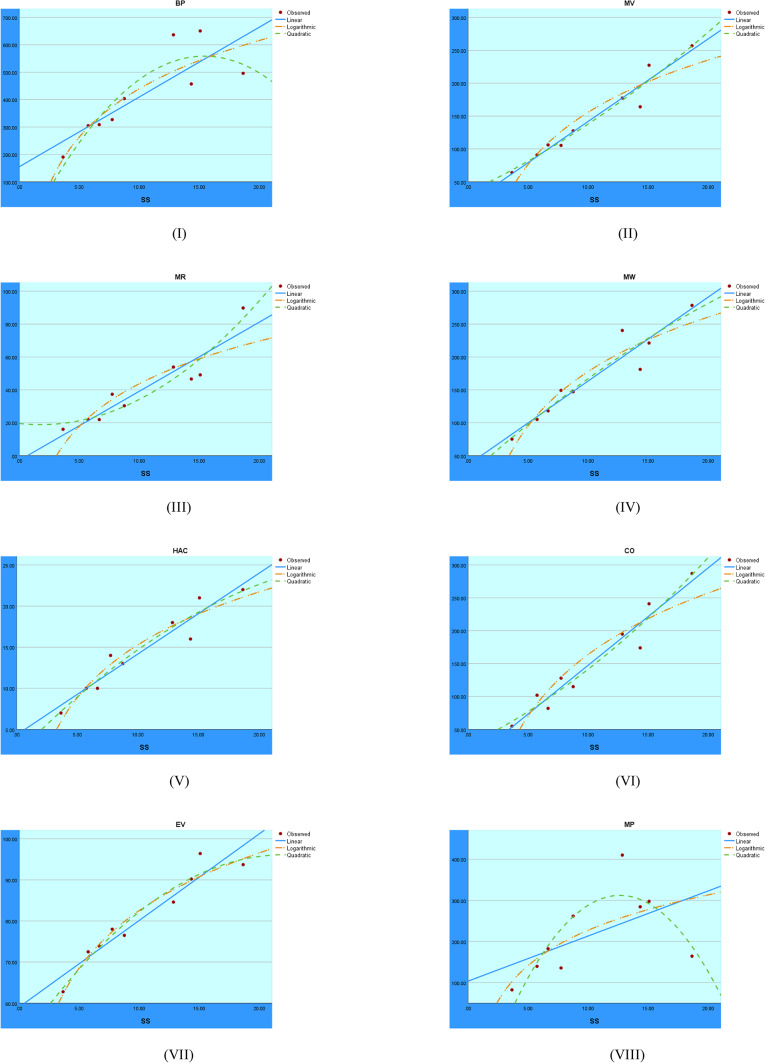
Visual representations of the regression analysis conducted on the SS index.

The regression models fitted to the SS index are shown in Fig. 6. The graphical comparison illustrates the prediction patterns of the logarithmic, quadratic, and linear models with respect to the SS index.

**Fig. 7:**
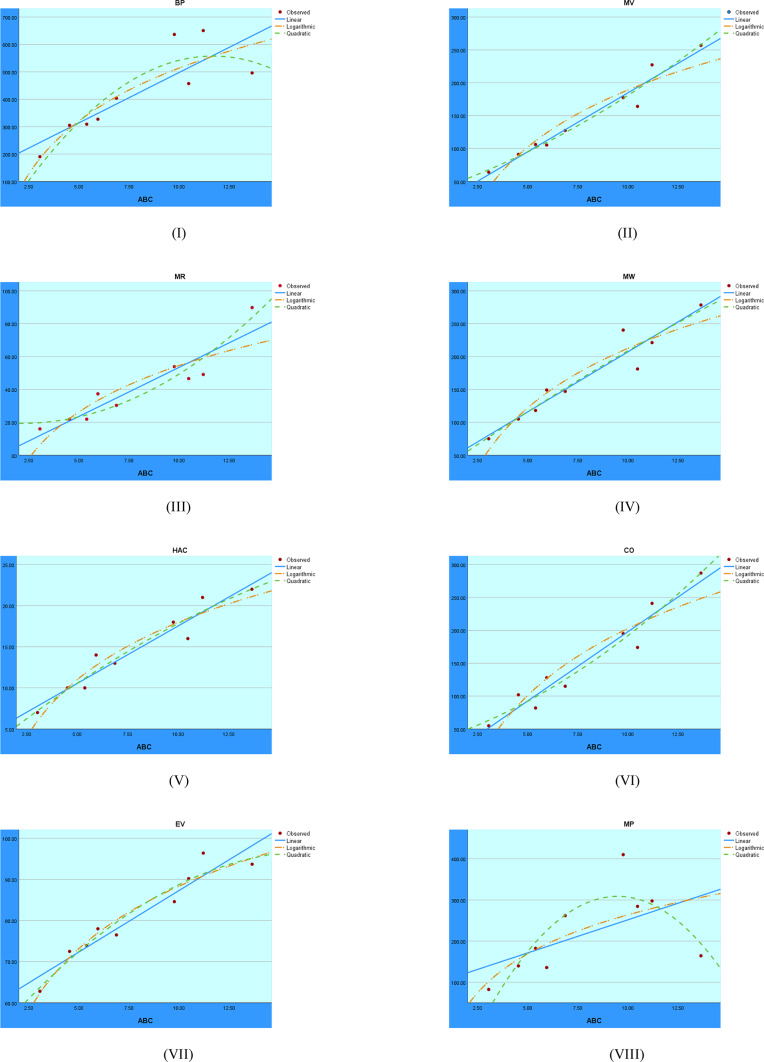
Regression analysis visuals for the ABC index.

The regression analysis findings of the ABC index are shown in Fig. 7. The graph contrasts how well the logarithmic, quadratic, and linear models capture the relationship between the outcome variable and the ABC index.

**Fig. 8:**
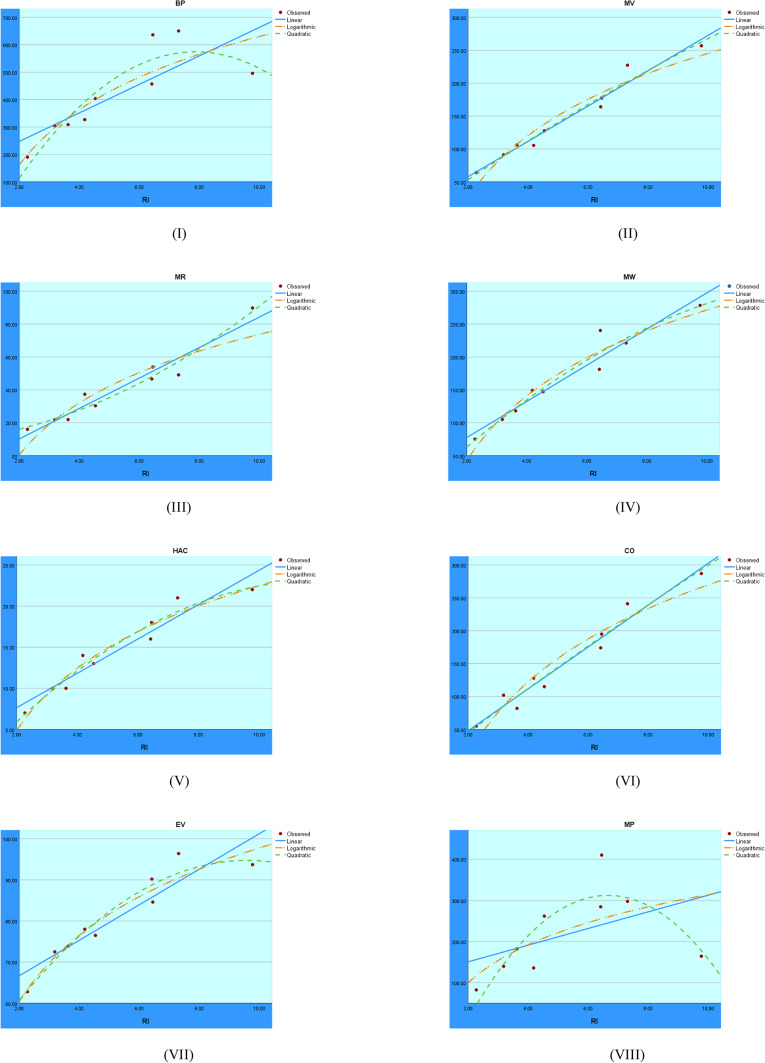
Regression analysis visuals for the RI index.

Figure 8 shows the visual regression results for the RI index. A comparison of the linear, quadratic, and logarithmic models provides insight into which model best fits the data for the RI index.

**Fig. 9:**
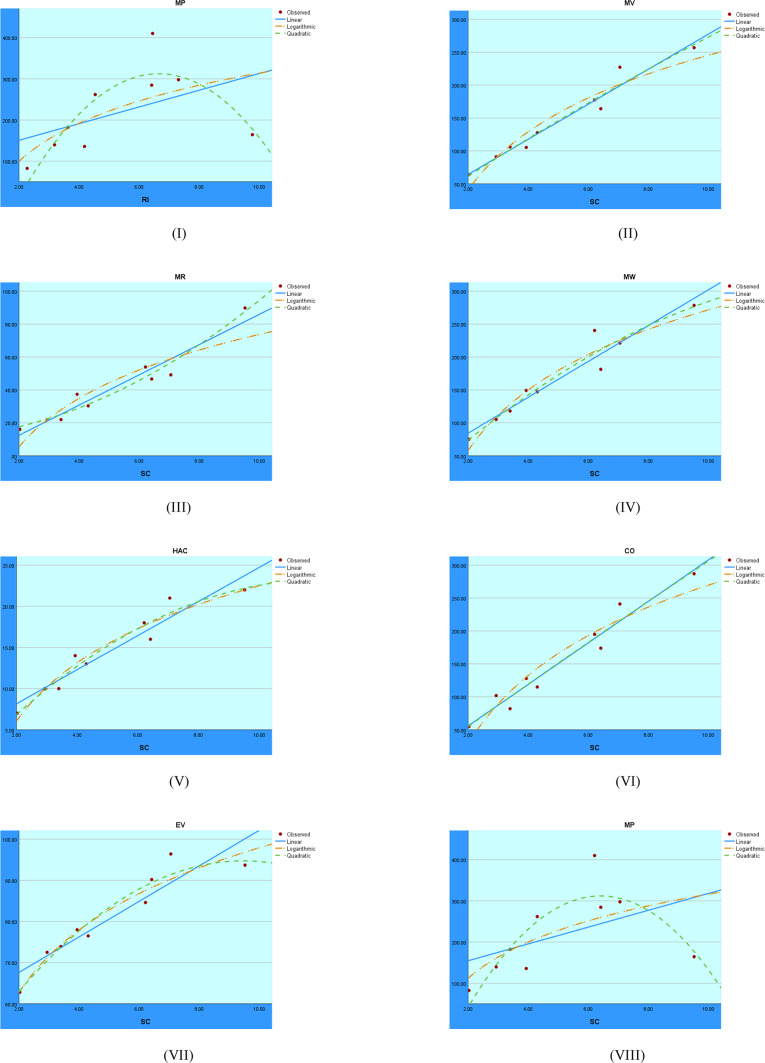
Regression analysis visuals for the SC index.

The regression analysis of the SC index using logarithmic, quadratic, and linear models is shown in Fig. 9. The plotted curves make it easier to understand the degree of fit and predictive power of each model.

**Fig. 10:**
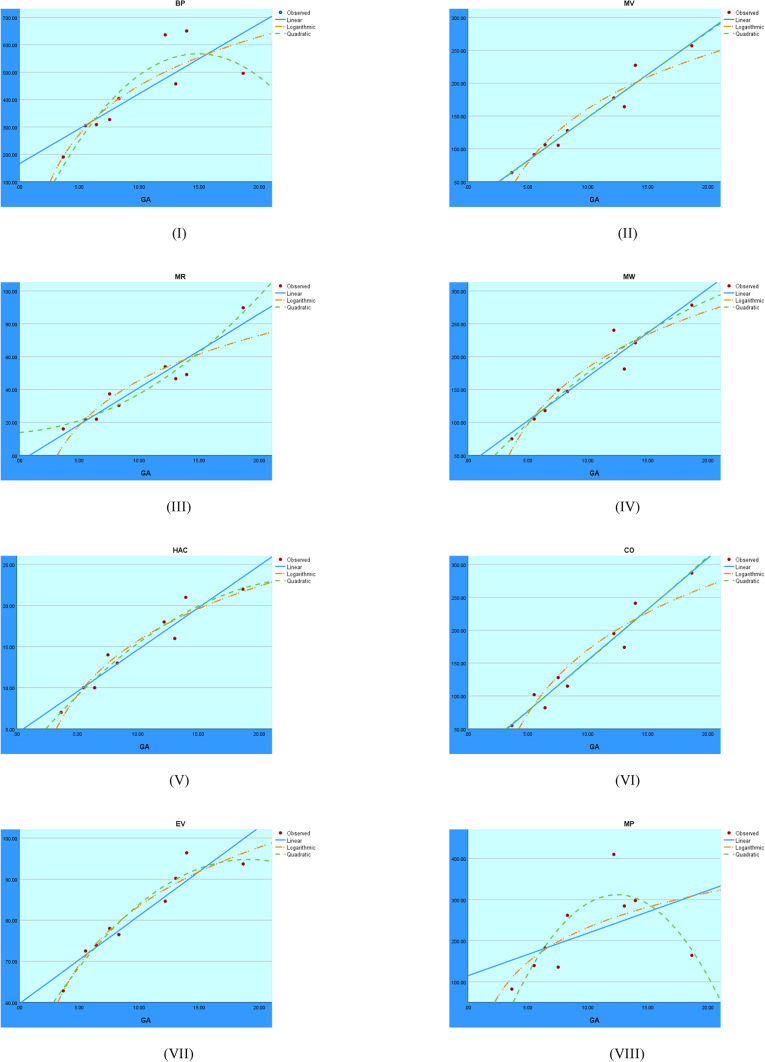
Visual representations of the regression analysis conducted on the GA index.

The regression results for the GA index are presented in Fig. 10. A visual comparison of the linear, quadratic, and logarithmic models’ agreement with the observed data for this index is provided in figure.

**Fig. 11:**
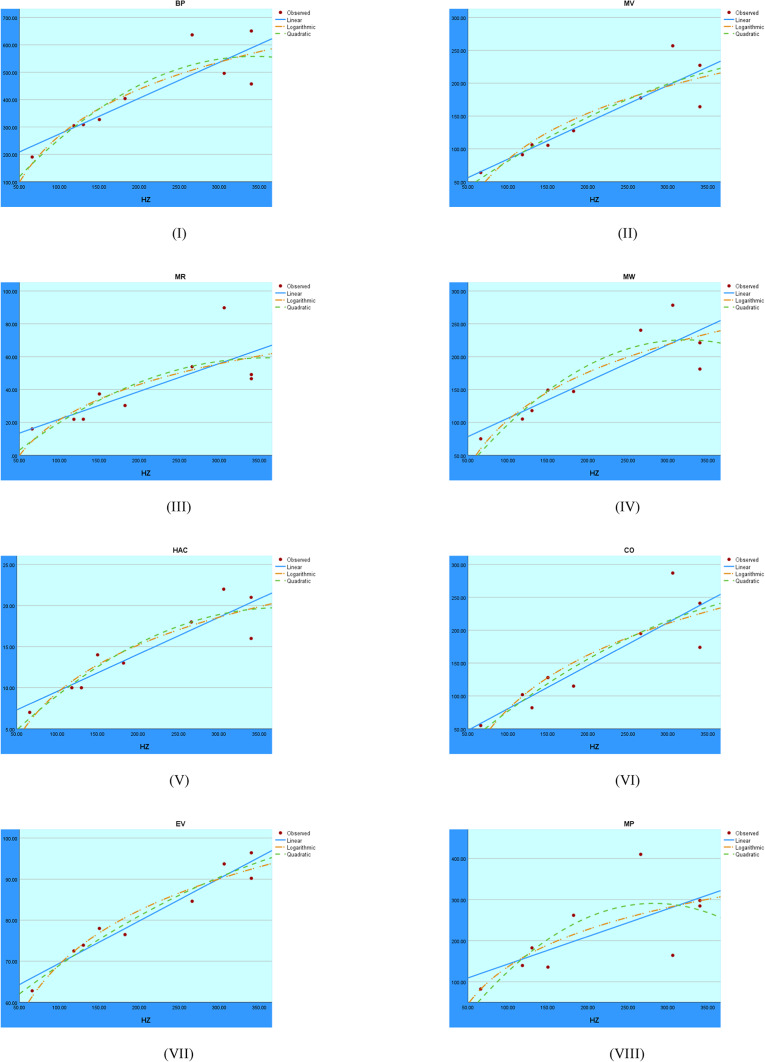
Visual representations of the regression analysis conducted on the HZ index.

The regression models applied to the HZ index are shown in Fig. 11. The trends shown aid in evaluating each model’s fit and ability to adequately explain the relationship with the target variable.

## Conclusion

This study demonstrated the reliability of topological indices as instruments for predicting the physical and chemical characteristics of different pharmaceutical substances. We found a strong relationship between the structural characteristics of the compounds and their properties using mathematical models, such as logarithmic, linear, and quadratic regression. Quadratic regression exhibited the highest predictive accuracy among all models tested. These findings imply that the use of labor-intensive and time-consuming laboratory experiments can be greatly reduced by employing computational approaches. Each of the physicochemical properties was individually compared to the predictive performance of the three regression models Linear, Quadratic and Logarithmic by choosing the best performing predictor (i.e., descriptor) within each model and then assessing the resulting coefficient of determination $R^2$. The comparative Tables from 18, 19, 20, 21, 22, 23, 24 and 25 show clearly that Quadratic regression model always had the highest $R^2$ values of all physicochemical properties, the BP, MV, MR, MW, HAC, CO, EV, and MP. Though the Linear and Logarithmic models gave moderately good correlations at least in certain instances, their limits of predictivity were lower than those that were derived using Quadratic modeling. This tendency leads to the idea that the use of second-order terms in defining the relationship between topological indices and physicochemical properties are more efficient compared to the use of linear and logarithmic transformations. Accordingly, the Quadratic model is more accurate in predictions and can hence be characterized as the most appropriate model that can be used to explain the structure-property relationships in this data. The future research might involve combining machine learning algorithms and topological indices to enhance the efficiency and scalability of predictive models of the complex drug properties. Moreover, the development of the indices in predicting a biological activity and poisoning profile represent a bridge into the promising field of improving early-stage drug discovery and safety analysis (Tables 19, 20, 21, 22, 23 and 24). In Tables 18 to Table 25, H is the harmonic index, RI is the Randic index, M2 is the second Zagreb index. These all indices have been defined in above mentioned definitions.

**Table 18:** BP: Model comparison (R$^2$).

Model	Best Predictor	$R^2$
Linear	HZ	0.760
Quadratic	**H**	**0.849**
Logarithmic	F, HZ	0.791
**Best model:** Quadratic (R$^2$ = **0.849**)

**Table 19:** MV: Model comparison (R$^2$).

Model	Best Predictor	$R^2$
Linear	RI	0.967
Quadratic	**RI**	**0.968**
Logarithmic	H	0.933
**Best model:** Quadratic (R$^2$ = **0.968**)

**Table 20:** MR: Model comparison (R$^2$).

Model	Best Predictor	$R^2$
Linear	H	0.955
Quadratic	**H**	**0.965**
Logarithmic	H	0.868
**Best model:** Quadratic (R$^2$ = **0.965**)

**Table 21:** MW: Model comparison (R$^2$).

Model	Best Predictor	$R^2$
Linear	–	–
Quadratic	**H**	**0.950**
Logarithmic	H	0.942
**Best model:** Quadratic (R$^2$ = **0.950**)

**Table 22:** HAC: Model comparison (R$^2$).

Model	Best Predictor	$R^2$
Linear	ABC	0.927
Quadratic	**H**	**0.957**
Logarithmic	RI	0.949
**Best model:** Quadratic (R$^2$ = **0.957**)

**Table 23:** CO: Model comparison (R$^2$).

Model	Best Predictor	$R^2$
Linear	RI	0.962
Quadratic	**RI**	**0.963**
Logarithmic	H	0.928
**Best model:** Quadratic (R$^2$ = **0.963**)

**Table 24:** EV: Model comparison (R$^2$).

Model	Best Predictor	$R^2$
Linear	M$_{2}$	0.955
Quadratic	**M**$_{2}$	**0.958**
Logarithmic	M$_{2}$	0.947
**Best model:** Quadratic (R$^2$ = **0.958**)

**Table 25:** MP: Model comparison (R$^2$).

Model	Best Predictor	$R^2$
Linear	–	–
Quadratic	**H**	**0.760**
Logarithmic	–	–
**Best model:** Quadratic (R$^2$ = **0.760**)		

## Data Availability

The datasets used and/or analysed during the current study available from the corresponding author on reasonable request.
